# Supplementary material for: Evaluating large language models for drafting emergency department encounter summaries
Source: PLOS Digit Health. 2025 Jun 17;4(6):e0000899. doi: 10.1371/journal.pdig.0000899 (PMC12173386; doi:10.1371/journal.pdig.0000899)
Supplement: S1 Fig — (DOCX) [file pdig.0000899.s001.docx]

** S1 Fig.** Histogram of original Emergency Medicine provider note length among the n = 100 sample of Emergency Department encounters randomly selected for GPT-3.5-turbo and GPT-4 summarization.
