# Supplementary material for: Evaluating large language models for drafting emergency department encounter summaries
Source: PLOS Digit Health. 2025 Jun 17;4(6):e0000899. doi: 10.1371/journal.pdig.0000899 (PMC12173386; doi:10.1371/journal.pdig.0000899)
Supplement: S2 Fig — (DOCX) [file pdig.0000899.s002.docx]

**S2 Fig.** Histogram of word counts of a) GPT-3.5-turbo and b) GPT-4 generated encounter summaries.
