# Supplementary material for: Evaluating large language models for drafting emergency department encounter summaries
Source: PLOS Digit Health. 2025 Jun 17;4(6):e0000899. doi: 10.1371/journal.pdig.0000899 (PMC12173386; doi:10.1371/journal.pdig.0000899)
Supplement: S3 Fig — (DOCX) [file pdig.0000899.s003.docx]

a)

b)

c)

**Figure S3.** Manual categorization of reviewer comments providing further details for each error subtype [a) Inaccuracy, b) Hallucination, and c) Clinical omission] among GPT-4-generated encounter summaries compared to the ground-truth, original Emergency Medicine provider note.
