# Supplementary material for: Evaluating large language models for drafting emergency department encounter summaries
Source: PLOS Digit Health. 2025 Jun 17;4(6):e0000899. doi: 10.1371/journal.pdig.0000899 (PMC12173386; doi:10.1371/journal.pdig.0000899)
Supplement: S1 Table — *p < 0.001 (Chi-squared test, χ² = 26.0). (DOCX) [file pdig.0000899.s005.docx]

|  | Number of labels with inter-reviewer agreement (%) | | |
| --- | --- | --- | --- |
|  | Inaccuracy | Hallucination | Omission |
| GPT-3.5-turbo summaries | 936/1000 (93.6%) | 917/1000 (91.7%) | 906/1000 (90.6%) |
| GPT-4 summaries | 979/1000 (97.9%) | 954/1000 (95.4%) | 931/1000 (93.1%) |
| **Total*** | **1915/2000 (95.8%)** | **1871/2000 (93.6%)** | **1837/2000 (91.9%)** |

**S1 Table.** Initial inter-reviewer agreement rates by error type, prior to consensus agreement. *p < 0.001 (Chi-squared test, χ² = 26.0).
