# Supplementary material for: Evaluating large language models for drafting emergency department encounter summaries
Source: PLOS Digit Health. 2025 Jun 17;4(6):e0000899. doi: 10.1371/journal.pdig.0000899 (PMC12173386; doi:10.1371/journal.pdig.0000899)
Supplement: S1 Text — Example GPT-3.5-turbo and GPT-4 generated encounter summaries and original Emergency Medicine note. (DOCX) [file pdig.0000899.s008.docx]

**S1 Text**

Below are three example cases consisting of a) the original Emergency Medicine note alongside b) the GPT-3.5-turbo-generated discharge summary and c) the GPT-4-generated discharge summary of that note.

*Some details in the deidentified original note may have been additionally modified to preserve patient confidentiality. **Any dates shown in the text have been artificially shuffled for de-identification.

**Example 1**

***Original Emergency Medicine Note:***

History Chief Complaint Patient presents with Hemorrhoids pain x 1 wk. Denies bleeding. H/o same several yrs ago. Denies abd pain, constipation. LBM yesterday, normal. History provided by: patient History limited by: nothing HPI 43yo hx HTN man with rectal pain x 1 week, progressively worsening rectal pain. November 30 sharp, worse with movement, not affected by bowel movements. Denies dysuria, abdo pain, N/V, blood in stool. Has hard BM consistently. Tried hemorrhoid cream without relief. Hemorrhoid 6 months ago, this pain is stronger. Allergies no known allergies Previous Medications No medications on file No past medical history on file. No past surgical history on file. History Social History Marital Status: N/A Spouse Name: N/A Number of Children: N/A Years of Education: N/A Occupational History Not on file. Social History Main Topics Smoking status: Not on file Smokeless tobacco: Not on file Alcohol Use: Not on file Drug Use: Not on file Sexually Active: Not on file Other Topics Concern Not on file Social History Narrative No narrative on file No family history on file. History, Medications and Nursing Notes were reviewed by ***** ***** ***** Review of Systems Review of Systems Constitutional: Negative for fever. Respiratory: Negative for cough and shortness of breath. Cardiovascular: Negative for chest pain. Gastrointestinal: Positive for constipation. Negative for nausea, vomiting, abdominal pain, diarrhea and blood in stool. Genitourinary: Negative for dysuria and hematuria. Musculoskeletal: Negative for back pain. All other systems reviewed and are negative. Physical Exam Triage Vital Signs: BP: 168/112 mmHg, Pulse - Palpated/Pleth: 80, *****Resp: 18, SpO2: 97 % Physical Exam Nursing note and vitals reviewed. Constitutional: He appears well-developed and well-nourished. No distress. HENT: Head: Normocephalic and atraumatic. Right Ear: External ear normal. Left Ear: External ear normal. Eyes: Conjunctivae and EOM are normal. Neck: Normal range of motion. Cardiovascular: Normal rate, regular rhythm and normal heart sounds. Exam reveals no gallop and no friction rub. No murmur heard. Pulmonary/Chest: Effort normal and breath sounds normal. No respiratory distress. He has no wheezes. He has no rales. He exhibits no tenderness. Abdominal: Soft. Bowel sounds are normal. He exhibits no distension. There is no tenderness. There is no rebound and no guarding. obese Genitourinary: 2-3cm firm dark purple hemorrhoid protruding from anus Musculoskeletal: Normal range of motion. Neurological: He is alert. Skin: Skin is warm and dry. Psychiatric: He has a normal mood and affect. His behavior is normal. Interpretations: Lab, Imaging, EKG & Rhythm Strip ED Course (Document differential diagnosis, ED treatment, response to treatment, reasons for choice of disposition, and whether further outpatient workup is needed or if patient is going to ***** or ICU) 43yo man with rectal pain x 1 week. H/o hemorrhoid 6 months ago. This feels similar but is stronger. ***** BMs consistently. Exam w/ thrombosed 2-3cm hemorrhoid. P: ***** Reassessment Removed clot from thrombosed hemorrhoid. ***** d/c w/ stool softener. Discussed use of sitz baths. Pt will f/u w/ PCP. Coding and Billing Info MDM ***** ***** *****, MD Resident 11/13/13

***GPT-3.5-turbo-generated discharge summary:***

This is a discharge summary for a 43-year-old male patient who presented to the Emergency Department with rectal pain due to hemorrhoids. The patient denied any bleeding and had a history of similar symptoms several years ago. He denied any abdominal pain, constipation, or dysuria. The patient had a normal bowel movement the day before presenting to the ED.

Upon examination, the patient had a thrombosed 2-3cm hemorrhoid protruding from the anus. His vital signs were stable, with a blood pressure of 168/112 mmHg, pulse rate of 80 beats per minute, respiratory rate of 18 breaths per minute, and oxygen saturation of 97%.

The patient's medical history, including medications and surgical history, was not available. There was no significant social history or family history provided.

The patient's review of systems was negative for fever, cough, shortness of breath, chest pain, nausea, vomiting, abdominal pain, diarrhea, blood in stool, dysuria, hematuria, and back pain.

Based on the history and physical examination findings, the patient was diagnosed with a thrombosed hemorrhoid. The treatment plan included removing the clot from the hemorrhoid and prescribing a stool softener. The patient was also advised to use sitz baths for symptom relief. The patient was discharged with instructions to follow up with their primary care physician.

No further outpatient workup was deemed necessary, and the patient did not require admission to the hospital or intensive care unit.

Signed,

***** ***** *****, MD

Emergency Department Physician

Date: 11/13/13

***GPT-4-generated discharge summary:***

Patient Information:

43-year-old male with a history of hypertension and previous hemorrhoids.

Chief Complaint:

Patient presented with worsening rectal pain over the past week, similar to a previous episode of hemorrhoids but more intense.

History of Present Illness:

The patient has been experiencing progressively worsening rectal pain over the past week, which is sharp and worsens with movement. The pain is not affected by bowel movements. The patient denies dysuria, abdominal pain, nausea, vomiting, and blood in stool. He has been having hard bowel movements consistently. He tried hemorrhoid cream without relief. He had a previous episode of hemorrhoids 6 months ago.

Physical Examination:

The patient appeared well-developed and well-nourished, with no signs of distress. Vital signs were stable. A 2-3cm firm dark purple hemorrhoid was noted protruding from the anus. The rest of the physical examination was unremarkable.

Treatment and Course in ED:

The patient was diagnosed with a thrombosed hemorrhoid. The clot was removed from the thrombosed hemorrhoid. The patient was discharged with a stool softener and advised to use sitz baths. He was instructed to follow up with his primary care physician.

Discharge Instructions:

The patient was advised to take the prescribed stool softener and to use sitz baths. He was instructed to follow up with his primary care physician.

Follow-up:

The patient is to follow up with his primary care physician.

**Example 2**

***Original Emergency Medicine Note:***

ED First Attending *****, ***** ***** ***** Chief Complaint Patient presents with Hemoptysis pt states hemoptysis x 1 day/also with epistaxis. Recent *****. -n/v/d/abd pain This is an independent visit and my billing provider is ***** ***** History provided by: the patient History limited by: nothing HPI 24 y.o. male presents with episode of epistaxis last night. Recent *****. Currently reports rhinorrhea and post nasal drip. Epistaxis after he "stuffed tissue" in his nares. Pt coughed up dry small amount of blood from nose bleed. He is not taking any medications currently. Denies bruising easily. No chest pain, dyspnea, headache, dizziness, or lightheadedness. No family history of coagulopathy. +smoker. Allergies Allergen Reactions Penicillins Rash Discharge Medication List as of *****/*****/***** *****:***** PM CONTINUE these medications which have NOT CHANGED Details albuterol (PROAIR HFA) 90 mcg/actuation metered dose inhaler Inhale 1 puff into the lungs every 4 (four) hours as needed for Wheezing or Shortness of Breath., Starting 12/14/2014, Until Discontinued, Normal diclofenac (VOLTAREN) 75 mg EC tablet Take 1 tablet (75 mg total) by mouth Twice a day., Starting 10/22/2013, Until Discontinued, Normal !! guaifenesin-codeine (GUAIFENESIN AC) 100-10 mg/5 mL liquid Take 5 mLs by mouth 2 (two) times daily as needed for Cough., Starting 05/30/2014, Until Discontinued, Print !! guaifenesin-codeine (GUAIFENESIN AC) 100-10 mg/5 mL liquid Take 5 mLs by mouth 2 (two) times daily as needed for Cough., Starting 05/30/2014, Until Discontinued, Print ibuprofen (ADVIL,MOTRIN) 600 mg tablet Take 1 tablet (600 mg total) by mouth 4 (four) times daily with meals and nightly., Starting 04/18/2016, Until Discontinued, Print naproxen (NAPROSYN) 500 mg tablet Take 1 tablet (500 mg total) by mouth 2 (two) times daily with meals., Starting 05/30/2014, Until Discontinued, Normal !! - Potential duplicate medications found. Please discuss with provider. Past Medical History: Diagnosis Date Asthma No past surgical history on file. Social History Social History Marital status: Single Spouse name: N/A Number of children: N/A Years of education: N/A Occupational History Not on file. Social History Main Topics Smoking status: Never Smoker Smokeless tobacco: Not on file Alcohol use Yes Comment: rare, 2x per month Drug use: No Sexual activity: Yes Partners: Female Birth control/ protection: Condom Other Topics Concern Not on file Social History Narrative Mr. ***** lives in ***** ***** with his mother. Also has a brother. ***** up in the ***** *****. He currently is doing warehouse work for ***** ***** but looking ***** ***** jobs, previously working for ***** *****. He is going to be employed at ***** ***** location either in the warehouse. Diet: eats servings a vegetables every other day or so Exercise: plays basketball, walks, active ***** ***** job Injury prevention: no biking or motorcycle, no dangerous hobbies, wears ***** Family History Problem Relation Age of Onset Congestive heart failure Maternal Grandmother 65 Hypertension Mother Hypertension Maternal Grandmother Other Maternal Grandmother Other Mother Diabetes Maternal Grandmother History, Medications and Nursing Notes were reviewed by ***** ***** Review of Systems Review of Systems Constitutional: Negative for chills, fatigue and fever. HENT: Positive for congestion, nosebleeds, postnasal drip, rhinorrhea, sinus pressure and sore throat. Negative for tinnitus and voice change. Respiratory: Positive for cough. Negative for shortness of breath and wheezing. Cardiovascular: Negative for chest pain. Musculoskeletal: Negative for myalgias. Skin: Negative for color change. Neurological: Negative for dizziness, light-headedness and headaches. Physical Exam Triage Vital Signs: BP: 149/88, Pulse - Palpated/Pleth: 97, Temp: 36.7 C (98.1 *****), *****Resp: 19, SpO2: 95 % Physical Exam Nursing note and vitals reviewed. Constitutional: He is oriented to person, place, and time. He appears well-developed and well-nourished. He does not appear ill. No distress. HENT: Head: Normocephalic and atraumatic. Right Ear: Tympanic membrane normal. Left Ear: Tympanic membrane normal. Nose: Rhinorrhea present. Epistaxis (scant amount of dry blood in left nare) is observed. Mouth/Throat: Uvula is midline, oropharynx is clear and moist and mucous membranes are normal. Neck: Trachea normal, normal range of motion and phonation normal. Cardiovascular: Normal rate, regular rhythm and normal heart sounds. Pulmonary/Chest: Effort normal and breath sounds normal. No respiratory distress. Neurological: He is alert and oriented to person, place, and time. Skin: Skin is warm and dry. He is not diaphoretic. No pallor. Psychiatric: He has a normal mood and affect. His speech is normal and behavior is normal. Interpretations: Lab, Imaging, EKG & Rhythm Strip CXR: negative acute ED Course (Document differential diagnosis, ED treatment, response to treatment, reasons for choice of disposition, and whether further outpatient workup is needed or if patient is going to ***** or ICU) ED Course Epistaxis, and Acute upper respiratory infection No active bleeding today follow up w pcp Return precautions discussed Reassessment Coding and Billing Info MDM ***** *****, NP 06/20/16 2332 ***** ***** *****, MD 06/23/16

***GPT-3.5-turbo-generated discharge summary:***

Patient: ***** *****

Date of Admission: 06/20/16

Date of Discharge: 06/23/16

Chief Complaint:

The patient presented to the Emergency Department with hemoptysis and epistaxis, as well as symptoms of nausea, vomiting, diarrhea, and abdominal pain.

History of Present Illness:

The 24-year-old male patient reported an episode of epistaxis the previous night, which was followed by rhinorrhea and post nasal drip. The epistaxis occurred after the patient "stuffed tissue" in his nares. He also experienced a small amount of dry blood coughed up from the nose bleed. The patient denied any chest pain, dyspnea, headache, dizziness, or lightheadedness. He had no family history of coagulopathy and reported being a smoker.

Allergies:

The patient has a known allergy to penicillins, which causes a rash.

Medication List:

The patient is currently taking the following medications:

- Albuterol (PROAIR HFA) 90 mcg/actuation metered dose inhaler: Inhale 1 puff into the lungs every 4 hours as needed for wheezing or shortness of breath.

- Diclofenac (VOLTAREN) 75 mg EC tablet: Take 1 tablet (75 mg total) by mouth twice a day.

- Guaifenesin-codeine (GUAIFENESIN AC) 100-10 mg/5 mL liquid: Take 5 mLs by mouth 2 times daily as needed for cough.

- Ibuprofen (ADVIL, MOTRIN) 600 mg tablet: Take 1 tablet (600 mg total) by mouth 4 times daily with meals and nightly.

- Naproxen (NAPROSYN) 500 mg tablet: Take 1 tablet (500 mg total) by mouth 2 times daily with meals.

Past Medical History:

The patient has a history of asthma but no past surgical history.

Social History:

The patient is single and lives with his mother and brother. He currently works in a warehouse but is looking for other job opportunities. He does not have any significant occupational history. The patient does not smoke or use drugs, but he does consume alcohol occasionally (2 times per month). He is sexually active with female partners and uses condoms for birth control/protection.

Family History:

The patient's maternal grandmother has a history of congestive heart failure, hypertension, and diabetes. The patient's mother also has a history of hypertension. Other family history details were not provided.

Review of Systems:

The patient denied chills, fatigue, and fever. He reported congestion, nosebleeds, postnasal drip, rhinorrhea, sinus pressure, sore throat, and cough. He did not experience shortness of breath, wheezing, chest pain, myalgias, dizziness, light-headedness, headaches, or any color changes on the skin.

Physical Examination:

The patient's vital signs were stable, with a blood pressure of 149/88 mmHg, pulse rate of 97 beats per minute, temperature of 36.7°C (98.1°F), respiratory rate of 19 breaths per minute, and oxygen saturation of 95%. The patient appeared well-developed and well-nourished, with no signs of distress. The physical examination revealed normal findings in the head, ears, nose, throat, neck, cardiovascular system, pulmonary/chest, neurological system, skin, and psychiatric status.

Interpretations:

A chest X-ray was performed, which showed no acute abnormalities.

ED Course:

The patient was diagnosed with epistaxis and acute upper respiratory infection. There was no active bleeding at the time of evaluation. The patient was advised to follow up with their primary care physician for further management. Return precautions were discussed with the patient.

Disposition:

The patient was discharged from the Emergency Department with instructions to follow up with their primary care physician. No further outpatient workup was deemed necessary.

Follow-up:

The patient should schedule an appointment with their primary care physician for further evaluation and management of their symptoms.

Signed,

***** *****, MD

Emergency Department Physician

***GPT-4-generated discharge summary:***

Patient Information:

24-year-old male

Chief Complaint:

Hemoptysis and epistaxis

History of Present Illness:

The patient presented with an episode of nosebleed (epistaxis) and coughing up a small amount of blood (hemoptysis) from a nose bleed. He also reported rhinorrhea and post nasal drip. The patient denied any chest pain, dyspnea, headache, dizziness, or lightheadedness. He has no family history of coagulopathy. He is a smoker.

Past Medical History:

The patient has a history of asthma. He has no past surgical history.

Medications:

The patient is advised to continue his current medications which include albuterol, diclofenac, guaifenesin-codeine, ibuprofen, and naproxen.

Physical Examination:

Upon examination, the patient was oriented to person, place, and time. He appeared well-developed and well-nourished. He did not appear ill and was not in distress. Examination of the head, ears, and neck was normal. Rhinorrhea was present and a scant amount of dry blood was observed in the left nostril. Cardiovascular and pulmonary examinations were normal. Neurological examination was normal. Skin was warm and dry. Psychiatric examination was normal.

Diagnosis:

Epistaxis and acute upper respiratory infection

Treatment and Recommendations:

The patient was advised to follow up with his primary care provider. He was given return precautions.

Discharge Medications:

The patient is to continue his current medications which include albuterol, diclofenac, guaifenesin-codeine, ibuprofen, and naproxen.

Follow-Up:

The patient is advised to follow up with his primary care provider.

**Example 3**

***Original Emergency Medicine Note:***

ED Attendings History Chief Complaint Patient presents with Eye Injury HPI ***** is a healthy 18 yo male who was working on his car 1-2 hours prior to presentation, and was on his back under the car when a piece of metal fell into his L eye. He could see it in the mirror and so came to the ED. He describes feeling a foreign body sensation, but no pain. He has not taken any meds. He denies any visual changes. He did not try to remove it himself. PMH: None Meds: none Allergies/Contraindications Allergen Reactions Amoxicillin Medical History History reviewed. No pertinent past medical history. Surgical History History reviewed. No pertinent surgical history. Social History: ***** ***** ***** ***** and will be attending UTI in December to learn to be a mechanic. Review of Systems Review of Systems Constitutional: Negative for fatigue and fever. HENT: Negative for rhinorrhea and sore throat. Eyes: Negative for pain and visual disturbance. Respiratory: Negative for cough and shortness of breath. Cardiovascular: Negative for chest pain. Gastrointestinal: Negative for constipation and diarrhea. Endocrine: Negative for polyuria. Genitourinary: Negative for difficulty urinating. Musculoskeletal: Negative for arthralgias. Skin: Negative for rash. Neurological: Negative for headaches. Hematological: Does not bruise/bleed easily. Physical Exam Triage Vital Signs: BP: 114/56, Heart Rate: 67, Temp: 36.4 C (97.5 *****), *****Resp: 18 Physical Exam Vitals and nursing note reviewed. Constitutional: General: He is not in acute distress. Appearance: Normal appearance. HENT: Head: Normocephalic and atraumatic. Comments: 4-5mm dark brown ***** observed beneath the L lower eyelid superficially on the cornea Nose: Nose normal. Eyes: Extraocular Movements: Extraocular movements intact. Pulmonary: Effort: Pulmonary effort is normal. Musculoskeletal: General: Normal range of motion. Cervical back: Normal range of motion. Skin: General: Skin is warm and dry. Capillary Refill: Capillary refill takes less than 2 seconds. Neurological: General: No focal deficit present. Mental Status: He is alert. Psychiatric: Mood and Affect: Mood normal. Behavior: Behavior normal. Initial Assessment (problem list and differential diagnosis) Well-appearing 18yo ***** with foreign body superficially in L lower eye on cornea. Mechanism of injury was foreign body falling into eye from above, not propelled and no pain present. Minimal irritation observed around foreign body. Seems simple to remove at bedside. Interpretations: Lab, Imaging, and ECG No labs or imaging reviewed Reassessment and Final Disposition Foreign body removed from L eye at bedside without difficulty. No corneal abrasion observed. See procedure note for full details. Discharged home with return precautions for pain in L eye or vision changes. ED Course as of 10/18/2229 Others' Documentation 10/18/2020 2206 18 yo male working on car something into his left eye has foreign body sensation |-------------------------| | | 10/18/20 | | | 2141 | |-------------------------| | BP: | *****/***** | | Pulse: | 67 | | BP Location:|Right upper arm| |-------------------------| Foreign body removal from surface of eye ***** negative ***** *****'***** ***** MD 10:21 PM [MB] ED Course User Index [*****] ***** *****'***** *****, MD Patient seen by: ***** *****, MD Pediatrics PGY-1 UCSF Benioff Children's Hospital ***** ***** *****, MD Resident 10/19/20 0240 ***** *****'***** *****, MD 10/20/20

***GPT-3.5-turbo-generated discharge summary:***

Patient: 18-year-old male

Chief Complaint: Eye Injury

History of Present Illness: The patient, a healthy 18-year-old male, was working on his car when a piece of metal fell into his left eye. He immediately came to the Emergency Department (ED) as he could see the foreign body in the mirror. He reported a foreign body sensation but no pain. He did not attempt to remove it himself. There were no visual changes or other symptoms.

Past Medical History: None

Medications: None

Allergies/Contraindications: No known allergies or contraindications

Social History: The patient is currently a student and will be attending UTI in December to learn to be a mechanic.

Review of Systems: Negative for fatigue, fever, rhinorrhea, sore throat, pain, visual disturbance, cough, shortness of breath, chest pain, constipation, diarrhea, polyuria, difficulty urinating, arthralgias, rash, and headaches. The patient does not bruise or bleed easily.

Physical Examination: The patient appeared normal and was not in acute distress. There was a 4-5mm dark brown foreign body observed beneath the left lower eyelid superficially on the cornea. The rest of the physical examination was unremarkable.

Assessment: Well-appearing 18-year-old male with a foreign body superficially in the left lower eye on the cornea. The mechanism of injury was a foreign body falling into the eye from above, without propulsion or pain. Minimal irritation was observed around the foreign body. The foreign body was successfully removed at the bedside without difficulty, and no corneal abrasion was observed.

Plan: The patient was discharged home with instructions to monitor for any pain in the left eye or changes in vision. No further lab work or imaging was deemed necessary.

Instructions: The patient should seek medical attention if he experiences any pain in the left eye or any changes in vision. He should follow up with his primary care physician if symptoms worsen or persist.

Follow-up: The patient was advised to follow up with his primary care physician if symptoms worsen or persist.

This discharge summary is based solely on the information provided in the note. No additional information was available.

***GPT-4-generated discharge summary:***

Patient Information:

18-year-old male with no significant past medical or surgical history.

Chief Complaint:

Eye injury due to a foreign body (piece of metal) falling into the left eye while working on a car.

History of Present Illness:

The patient presented to the ED after a piece of metal fell into his left eye while he was working on his car. He reported a sensation of a foreign body in the eye but denied any pain or visual changes. He did not attempt to remove the foreign body himself.

Physical Examination:

On examination, a 4-5mm dark brown foreign body was observed superficially on the cornea of the left eye. The patient was not in acute distress and had normal appearance. Extraocular movements were intact. No other abnormalities were noted.

Treatment and Course in the ED:

The foreign body was successfully removed from the left eye at the bedside without difficulty. No corneal abrasion was observed.

Discharge Instructions:

The patient was discharged home with instructions to return to the ED if he experiences pain in the left eye or any changes in vision.

Follow-up:

No specific follow-up was mentioned in the note.
