# Supplementary material for: Evaluating large language models for drafting emergency department encounter summaries
Source: PLOS Digit Health. 2025 Jun 17;4(6):e0000899. doi: 10.1371/journal.pdig.0000899 (PMC12173386; doi:10.1371/journal.pdig.0000899)
Supplement: Attachment — Submitted filename: Response to Reviewers - FINAL.docx [file pdig.0000899.s010.docx]

**Reviewer #1:**

While this may have been practical for manual review, it limits statistical power and generalizability of findings. More robust approach would involve larger sample size with stratified random sampling to ensure adequate representation across different types of ED encounters, conditions, and complexity levels.

- Response: We thank the reviewer for their comment. We conducted a random sample of 100 encounters selected from our entire cohort of 202,059 ED visits. This sample size was chosen to reflect an appropriate balance between selecting a sufficiently diverse range of admissions/patient presentations while simultaneously limiting the burden of manual review by reviewers (our reviewers spent 100s of hours examining each summary for errors). We have noted this in the Limitations section: ‘*Second, due to the time and labor-intensive process of manual expert review, we included 100 randomly selected ED encounters in our sample, which may limit generalizability across different types of patient demographics and presenting symptoms’* (lines 329 to 332).
- Compared to the handful of other studies in the literature that have reported text summarization for discharge narratives, our sample size is at the upper limit. For example, Hegselmann (<https://arxiv.org/abs/2402.15422>) had a sample size of 100, Ellershaw (<https://openreview.net/pdf?id=1kDJJPppRG>) had a sample size of 53, and Hartman (<https://pubmed.ncbi.nlm.nih.gov/37639624/>) had a sample size of only 25.
- While a larger sample size may lead to a marginal increase in generalizability, the additional burden of review to facilitate this would be substantial and is outside the scope of this study. We agree that future studies should explore stratified or larger samples, potentially using automated methods to assist in scalable evaluation.

Demographic representation in study presents another important limitation. As acknowledged by authors, random predominantly consisted of White, Asian, or Black/African American patients, with limited representation of other minority groups. To address this, researchers should implement purposive sampling strategies to ensure adequate representation across all demographic groups.

- Response: We agree with the reviewer about the importance of acknowledging the limited representation of other minority groups – it is for this reason we have included a statement on this in the Limitations section of the study (lines 332 to 335). However, conducting stratified sampling across all eight race/ethnicity groups (White, Asian, Black/African American, Latinx, Other, Native Hawaiian/Other Pacific Islander, Southwest Asian and North African, Unknown/Declined) at sufficient sample sizes to support meaningful comparisons in LLM performance would require a substantially larger dataset and review effort, which is beyond the scope and resources of the current study. Nevertheless, we recognize the importance of evaluating equity in LLM performance and are actively considering this for future work.

Single-institution focus of study, examining notes only from UCSF ED, significantly limits its generalizability. Different institutions have varying documentation styles, EHR systems, and patient populations. Multi-center study including diverse healthcare settings would provide more comprehensive insights into LLM performance across different clinical environments and documentation practices.

- Response: We appreciate the reviewer’s point and agree that evaluating LLM performance across diverse healthcare settings is an important future direction. At the same time, we believe that establishing a strong foundation through single-institution studies is equally critical—particularly given the limited literature assessing LLMs on real-world clinical notes. Conducting this work in a single center allows for careful control over note structure, documentation style, and review process, enabling a focused and rigorous evaluation of model performance. While multi-center studies will be essential for assessing generalizability, they require considerable coordination and resources. To our knowledge, no multi-center studies of this nature have yet been published. In the context of the growing deployment of LLMs in clinical practice—often without thorough validation—we believe our study offers a timely and meaningful contribution to the field.
- We have commented on this in the Discussion: ‘*Future work should consider exploring the use of these models in multi-center studies across diverse patient populations.’* (lines 336-337).

Study's reviewer pool was limited to emergency medicine residents with one attending physician for arbitration. This narrow perspective might miss important aspects that different healthcare providers would value in discharge summaries.

- Response: The primary goal of the evaluation in this study was to identify errors in the LLM-generated summary, compared against the original Emergency Medicine note as reference. Given that a) Emergency Medicine residents are typically the personnel writing these notes, and b) the task of error identification is a comparatively simple task that does not require significant clinical expertise (i.e., the EM resident reviewers are more than capable of finding errors), we believe that this was an appropriate level of expertise for this study’s evaluation.
- In addition to this, we followed the widely-accepted best practice of two reviewers following a standard protocol to review each summary, with disagreements resolved by a 3^rd^ party (attending) adjudicator.

While study reports basic agreement rates, it lacks formal statistical analysis of inter-rater reliability. Including kappa statistics or other formal measures would provide more rigorous evidence of consistency and reliability of error identification process. Additionally, categorization of errors, particularly "omissions," appears somewhat subjective. Developing more standardized error classification framework with explicit criteria would improve reproducibility of findings.

- Response: We have reported inter-reviewer agreement rates (before consensus agreement) in Supplementary Table 1, with 95.8%, 93.6% and 91.9% agreement across Inaccuracy, Hallucination, and Omission errors, respectively. We, a priori, elected to use this approach over kappa statistics because our evaluation involved sparse and unbalanced outcomes – that is, most summaries contained only a small number of specific error types, and some categories were infrequently observed. In such settings, Cohen’s kappa can produce misleadingly low values even when raw agreement is high, due to the so-called “kappa paradox”, where skewed marginal totals reduce the statistic’s value despite strong consensus. For example, when the vast majority of summaries do not contain a particular type of error, kappa may underestimate true agreement. Given these limitations, we opted to calculate and report direct agreement rates, which we believe offer a clearer and more intuitive representation of reviewer consistency in this context.
- Categorization of errors was performed by each reviewer following a detailed, pre-defined classification framework specified in the Study Protocol (Supplementary File 1). This rigorous approach is reflected by our inter-reviewer agreement rates of over 90%, demonstrating reproducibility of findings.
- We acknowledge that there may be higher subjectivity of ‘Omissions’ reporting – and, indeed, we have commented on this in the Discussion – ‘*This reflects the subjective nature of classifying clinical omissions, where the inclusion of different clinical details may depend on the preference of the discharging clinician*’. However, even here, we believe this phenomenon had limited effect on the reproducibility of our findings given that inter-reviewer agreement rates for omissions remained above 90%.

Prompt engineering aspect of study appears limited, using single basic prompt without optimization. More comprehensive approach would involve systematic testing of different prompt variations and include few-shot learning examples to optimize LLM performance. This could significantly improve quality of generated summaries and provide valuable insights into best practices for implementing these systems in clinical settings.

- Response: We agree that evaluation of more advanced prompt variations and few-shot learning is an important avenue for future work. However, we believe that understanding the zero-shot capability of these models is just as important and a necessary foundation on which future research is built. We have highlighted this in the Discussion (lines 290 to 293): *‘It is possible that, with either dedicated prompt engineering or the addition of few-shot examples during future prompting, clinician-specific preferences of what information ought to be included in each encounter summary may be incorporated to address this.’*

While it identifies various types of errors, it doesn't evaluate their potential impact on patient care or quantify efficiency gains versus time needed for human verification. Including severity ratings for errors and analyzing practical implications of implementing LLM-based documentation tools would provide crucial information for healthcare organizations considering adoption of these technologies.

- Response: We thank the reviewer for this suggestion. We agree that evaluating severity ratings for errors is an important contribution and we have therefore **conducted a post-hoc analysis** of the potential for harm of each error identified in the GPT-4-generated summaries, using a modified version of the AHRQ Common Format Harm Scale adapted to reflect the potential for harm. We have added details on this to the Methods (lines 158 to 168), Results (lines 220 to 229) and Discussion (lines 261 to 263), finding that *‘the mean potential harmfulness score across all errors was 0.57 (SD 1.11) out of 7 on the adapted AHRQ Common Format Harm Scale. Stratified by error type, inaccuracy errors (mean 0.70, SD 1.25) had the highest potential for harm, followed by omission (0.67, SD 1.08) and hallucination (0.42, SD 1.11) errors’* and demonstrating that overall, errors had low potential for harm (0.57 out of 7).
- We agree that evaluating the practical efficiency gains of deploying LLM-based documentation tools will be important as LLMs begin to be used in clinical practice. However, as is common in the extant literature, evaluation of the ability of LLMs (or any new technology) at a given task and the consequent effects of deploying these technologies in clinical practice are separate entities that warrant separate studies. In this study, we performed an evaluation of the ability of LLMs to generate encounter summaries; we consider an examination of the practical implications of LLM use for discharge summarization to be outside the scope of this study.

Including multiple LLM versions and vendors, and evaluating performance changes across model iterations would provide more comprehensive understanding of technology's capabilities and limitations. This would also help healthcare organizations make more informed decisions about which systems might best suit their needs.

- Response: We thank the reviewer for this comment, however the evaluation of multiple vendors to allow healthcare organizations to make informed decisions about which systems best suit their needs is outside the scope of this study. Instead, the aim of this study was to investigate the performance of LLMs in a particular use case – the generation of ED encounter summaries – and evaluate the prevalence and type of errors across each section of the summary.

Starting with pilot study incorporating some of these improvements before conducting larger multi-center study would allow for refinement of methodology and identification of potential implementation challenges.

- Response: As discussed above, while these are important suggestions for future studies, we believe that they are outside the scope of this study.

**Reviewer #2: Review of "Evaluating Large Language Models for Drafting Emergency Department Encounter Summaries".**

This article addresses a highly relevant topic given the increasing use of large language models in healthcare. Electronic health record documentation is a significant burden on medical professionals, and leveraging LLMs to streamline this process is promising.

- Response: We thank the reviewer for their positive feedback.

However, concerns remain regarding their appropriate deployment and potential risks.

- Response: We thank the reviewer for raising these concerns, which we have responded to point-by-point, below.

1) The article specifically evaluates GPT-3.5 and GPT-4. It would be helpful to clarify the rationale for selecting these models over other commercially and open source available LLMs. Were they chosen based on prior benchmarking, accessibility, or specific performance attributes relevant to EHR summarization?

- Response: We thank the reviewer for pointing this out. GPT-3.5-turbo and GPT-4 were the latest state-of-the-art models available when we first planned the study and performed LLM summarization of the encounter notes.
- We have added this explanation of the choice of models to the Methods section: ‘*Although updated models have since been released, GPT-3.5-turbo and GPT-4 were the latest state-of-the-art models available at the time of study inception’* (lines 129 to 131).

2) The study sets the model temperature to 0 to encourage more deterministic outputs. However, GPT models do not default to this setting. Could the authors elaborate on why this specific temperature was chosen and whether alternative values were considered? Exploring different temperatures might provide insights into potential trade-offs between consistency and adaptability in the generated summaries.

- Response: We set model temperature to a value of 0 to facilitate reproducibility of LLM summarization, as a temperature of 0 results in the most deterministic outputs. This is an important step in ensuring scientific reproducibility and is widely accepted among the clinical NLP community – e.g: <https://jamanetwork.com/journals/jamanetworkopen/fullarticle/2818387>, <https://www.nature.com/articles/s41467-024-52415-1>. Moreover, even for tasks that do not require deterministic outputs for reproducibility, increasing evidence suggests that different temperatures do not have significant differences on overall results (e.g. see the appendix of this paper: <https://www.thelancet.com/journals/landig/article/PIIS2589-7500(23)00225-X/fulltext>).

3) It would strengthen the results section to include an analysis of whether identified errors had the potential to lead to adverse or dangerous clinical outcomes. Given that errors in EHR summarization can pose clinical risks, it would be beneficial to extend the discussion about potential solutions or mitigation strategies.

- Response: We thank the reviewer for this suggestion. As discussed above, we agree that analyzing whether errors had the potential to lead to adverse/dangerous clinical outcomes is an important consideration and we have therefore conducted a **post-hoc analysis** of the potential for harm of each error, using a modified version of the AHRQ Common Format Harm Scale adapted to reflect the potential for harm. We have added details on this to the Methods (lines 158 to 168), Results (lines 220 to 229) and Discussion (lines 261 to 263), finding that *‘the mean potential harmfulness score across all errors was 0.57 (SD 1.11) out of 7 on the adapted AHRQ Common Format Harm Scale. Stratified by error type, inaccuracy errors (mean 0.70, SD 1.25) had the highest potential for harm, followed by omission (0.67, SD 1.08) and hallucination (0.42, SD 1.11) errors’* and demonstrating that overall, errors had low potential for harm (0.57 out of 7).

4) The post hoc analysis might be better suited within the results section rather than in the discussion.

- Response: We thank the reviewer for this suggestion. We had originally considered including the post-hoc analysis (where we excluded any hallucinations of follow up instructions or of redacted information and then re-calculated the proportion of GPT-4 generated summaries considered error-free across all domains) in the Results rather than Discussion. However, because 1) this analysis had not been part of the initial, pre-specified Protocol and 2) we did not want to misrepresent the final tallied error-rate and/or to be seen to artificially ‘improve’ the error-free rate reported in the Results, we decided to simply highlight it as part of the commentary of the Discussion.
- For these reasons, it is our preference to retain this section in the Discussion, but we are open to moving it to Results if the Editor feels strongly.

5) Figure 2: The vertical line in the middle of the bars for different models could be misleading. It might imply an artificial boundary, affecting interpretation. Instead of grouping by model, an alternative visualization could categorize errors by type, allowing for clearer insights into which errors are most prevalent across models.

- We appreciate the reviewer’s suggestion and understand the concern. In Figure 2, we opted to present results for GPT-3.5-turbo and GPT-4 side-by-side to allow readers to directly compare the proportion of summaries with one or more errors across the three error types (Inaccuracy, Hallucination, Omission). The color coding was intended to enhance visual clarity between models. That said, we are happy to revise the figure layout if the Editor feels an alternative presentation would better support interpretation.

6) Figure 3: Similar to Figure 2, the visualization could be adjusted for better readability. The combination of horizontal and vertical lines, along with count values, makes comparisons difficult. The GPT-4 white box label overlaps with the first plot, which should be adjusted for clarity.

- We thank the reviewer for pointing out that the white box label overlaps with the first plot and we have adjusted this accordingly.
- We included horizontal and vertical lines to help readers identify the error count for each domain (i.e., the reader can use the horizontal lines to help read across from any ‘bar’ to the Count labeled on the y-axis). We would be happy to change the Figure at the Editor’s discretion.

7) Figure 2 and 3: The graphs are not showing the proportion of cases with more than one error and may mislead to wrong assumptions.

- We thank the reviewer for this comment. We believe there may be some misunderstanding, and we’d be grateful for clarification.
- Figure 2 displays the proportion of summaries containing at least one error in each category (Inaccuracy, Hallucination, or Omission), as indicated in the figure legend.
- Figure 3, on the other hand, provides a breakdown of where those errors occurred within the structure of the discharge summary (e.g., Presenting Complaint, Physical Exam). Our intent was to help readers identify which sections are more prone to specific types of errors. That said, we are happy to revise or clarify these figures further if the Editor feels it would improve interpretability.
